# Supplementary material for: Exploiting oxidative phosphorylation to promote the stem and immunoevasive properties of pancreatic cancer stem cells
Source: Nat Commun. 2020 Oct 16;11:5265. doi: 10.1038/s41467-020-18954-z (PMC7567808; doi:10.1038/s41467-020-18954-z)
Supplement: Supplementary file 1 — Supplementary Information [file 41467_2020_18954_MOESM1_ESM.pdf]

Exploiting Oxidative Phosphorylation to Promote the Stem and Immunoevasive Properties of Pancreatic Cancer Stem Cells

Sandra Valle, Sonia Alcalá, Laura Martin-Hijano, Pablo Cabezas-Sainz, Diego Navarro, Edurne Ramos Muñoz, Lourdes Yuste, Kanishka Tiwary, Karolin Walter, Laura Ruiz-Cañas, Marta Alonso-Nocelo, Juan A. Rubiolo, Emilio González-Arnay, Christopher Heeschen, Laura Garcia-Bermejo, Patrick C. Hermann, Laura Sánchez, Patricia Sancho, Miguel Ángel Fernández-Moreno, Bruno Sainz, Jr.

Supplementary Table 1. Antibodies

1ª Abs-Epitope	Source	Dilution	Application	Manufacturer
α-hu-CD133/1-APC	Mouse monoclonal	1:10	FC	Miltenyi Biotec (Cat no. 130-090-826)
α-hu-CD24-PEVio700	Mouse monoclonal	1:10	FC	Miltenyi Biotec (Cat no.130-108-381)
α-hu-CXCR4-PE	Mouse monoclonal	1:10	FC	Miltenyi Biotec (Cat no.130-103-798)
α-hu-TEM8	Mouse monoclonal	1:50	FC	Abcam (Cat no. ab21270)
α-hu-PD-L1-Alexa700	Mouse monoclonal	1:10	FC	BioLegend (Cat no. 329952)
α-hu-CD47-APC	Mouse monoclonal	2,5:50	FC	Miltenyi Biotec (Cat no 130-101-407)
α-hu-CD155-APC	Mouse monoclonal	1:10	FC	Miltenyi Biotec (Cat no 130-105-906)
α-hu-CD206-FITC	Mouse monoclonal	2,5:50	FC	Miltenyi Biotec (Cat no 130-095-13)
α-hu-ULBP2/5/6	Mouse monoclonal	1:50	FC	R&D systems (Cat no. MAB1298)
α-hu-CD90-APC	Mouse monoclonal	2,5:50	FC	Life Technologies (A15726)
α-β-ACTIN	Mouse monoclonal	1:5000	WB	ThermoFisher (Cat no.MA1-140)
α-GAPDH	Mouse monoclonal	1:5000	WB	ThermoFisher (Cat no.MA5-15738)
α-hu-LC3BI/II	Rabbit monoclonal	1:500/1:100	WB/IF	Sigma (Cat no. L7543)
α-hu-LAMP-1 (H43A)	Mouse monoclonal	1:100	IF	Santa Cruz (Cat no. sc-20011)
α-hu-PARKIN	Rabbit monoclonal	1:500/1:200	FC/IF	ThermoFisher (Cat no.PA5-13398)
α-hu-SSEA4-APC	Mouse monoclonal	1:50	FC	BioLegend (Cat no. 330418)
α-hu-CD44-PE	Mouse monoclonal	1:50	FC	Becton Dickinson (Cat no. 550989)
TOM20	Mouse monoclonal	1:200	IF	Santa Cruz (Cat no. sc-17764)
α-ms-CD45-FITC	Rat monoclonal	1:100	FC	BDbioscience (Cat no. 553080)
α-ms-CD11b-PerCP Cy5.5	Rat monoclonal	1:200	FC	TONBO (Cat no. 65-0112-U100)
α-ms-F4-80-PE	Recombinant human	1:100	FC	Miltenyi Biotec (Cat no 130-102-422)
α-hu- Cytokeratin 19	Mouse monoclonal	1:2000	IHC	Abcam (Cat no ab9221)

2ª Abs-Epitope	Source	Dilution	Application	Manufacturer
α-mouse-HRP	Sheep	1:5000	WB	Amersham (Cat no. NA9310-1ML)
α-rabbit-HRP	Donkey	1:5000	WB	Amersham (Cat no. NA9340-1ML)
α-mouse Alexa 647	Goat	1:500	IF	Invitrogen (Cat. no. A32728)
α-rabbit Alexa 555	Goat	1:500	IF/FC	Invitrogen (Cat. no. A32732)
α-Mouse IgGs-HRP	Goat	1:5000	IHC	DAKO (Cat no. P0447)

WB = Western blot; FC = Flow cytometry; IF = Immunofluorescence; IHC = Immunohistochemistry

Supplementary Table 2. RTqPCR human primer sequences

Gene	Application	Primer sense	Primer antisense
β-Actin	RTqPCR	GCGAGCACACGAGCCTCGCCTT	CATCATCCATGGTGAGCTGGCGG
Klf4	RTqPCR	ACCCACACAGGTGAGAAACC	ATGTGTAAGGCGAGGTGGTC
MT-RNR1	qPCR	CCACGGGAAACAGCAGTGAT	CTATTGACTTGGGTTAATCGTGTGA
Oct3/4	RTqPCR	CTTGCTGCAGAAGTGGGTGGAGGAA	CTGCAGTGTGGGTTTCGGGCA
Sox2	RTqPCR	AGAACCCCAAGATGCACAAC	CGGGGCCGGTATTTATAATC
Slug	RTqPCR	GGGGAGAAGCCTTTTTCTTG	TCCTCATGTTTGTGCAGGAG
Snail	RTqPCR	CTCCCTGTCAGATGAGGAC	CCAGGCTGAGGTATTCCTTG
Vimentin	RTqPCR	GAGAACTTTGCCGTTGAAGC	GCTTCCTGTAGGTGGCAATC
Zeb1	RTqPCR	CCAGGTGTAAGCGCAGAAA	CCACAATATGCAGTTTGTCTTCA
PGC1α	RTqPCR	TGACTGGCGTCATTCAGGAG	CCAGAGCAGCACACTCGAT
ABCG2	RTqPCR	CCGCTGGAATGCAAAATAG	CCATACGAACAGCTCCACA
Nanog	RTqPCR	TGAACCTCAGCTACAAACAGGTG	AACTGCATGCATGCAGGACTGCAGAG
Sox9	RTqPCR	ACGCACATCTCCCCCAACGC	GCATTGCCCCGAGTGCTCGCC
Cdh1	RTqPCR	TGCCCAGAAAATGAAAAAGGC	GTGTATGTGGCAATGCGTTC
NRF2	RTqPCR	AGGAGGAGGAAGTGGAGGGACTGA	ACATTCAGCTGGCGCGTAGGTT
NFκB	RTqPCR	CTCGCCACCCGGCTTCAGAAT	AAGGTATGGGCCATCTGTTGGCAG
ATG5	RTqPCR	AAGCAACTCTGGATGGGATTGC	AAAGGTCTTTCAGTCGTTGTCTGAT
NDUFA9	RTqPCR	AGTGGAGCGGATGCACATCACA	GACGGTCTTGGCCGGCTTCA
MT-COI	RTqPCR	CTCTTCGTCTGATCCGTCCT	ATTCCGAAGCCTGGTAGGAT
MT-RNR2	RTqPCR	CCCGATGGTGCAGCCGCTATTA	TCATTTACGGGGGAAGGCGCT
PARKIN	RTqPCR	CCGGCTGACCAGTTGCGTGT	GTCACAATTCTGCACAGTCCAGTCA
GARS	RTqPCR	GCACACACTGTCTCTGCCTG	CCGAATGATGAAGGAGGAAG
MKLN1	RTqPCR	GTTCTCCTTGTGAAATCCGC	TTCTTGCATGAGCAAACTGG
SFPQ	RTqPCR	TCGTACTGTTAGGCCCTTGG	AACCTTGCATGAAGAGCACC
HOTAIR	RTqPCR	GGCGGATGCAAGTTAATAAAAC	TACGCCTGAGTGTTCACGAG
MSI-1	RTqPCR	CTTCGGCCAGTTCGGGGAGG	TGACGAAGCCGAAACCCCTGG

RTqPCR = Real-Time quantitative PCR

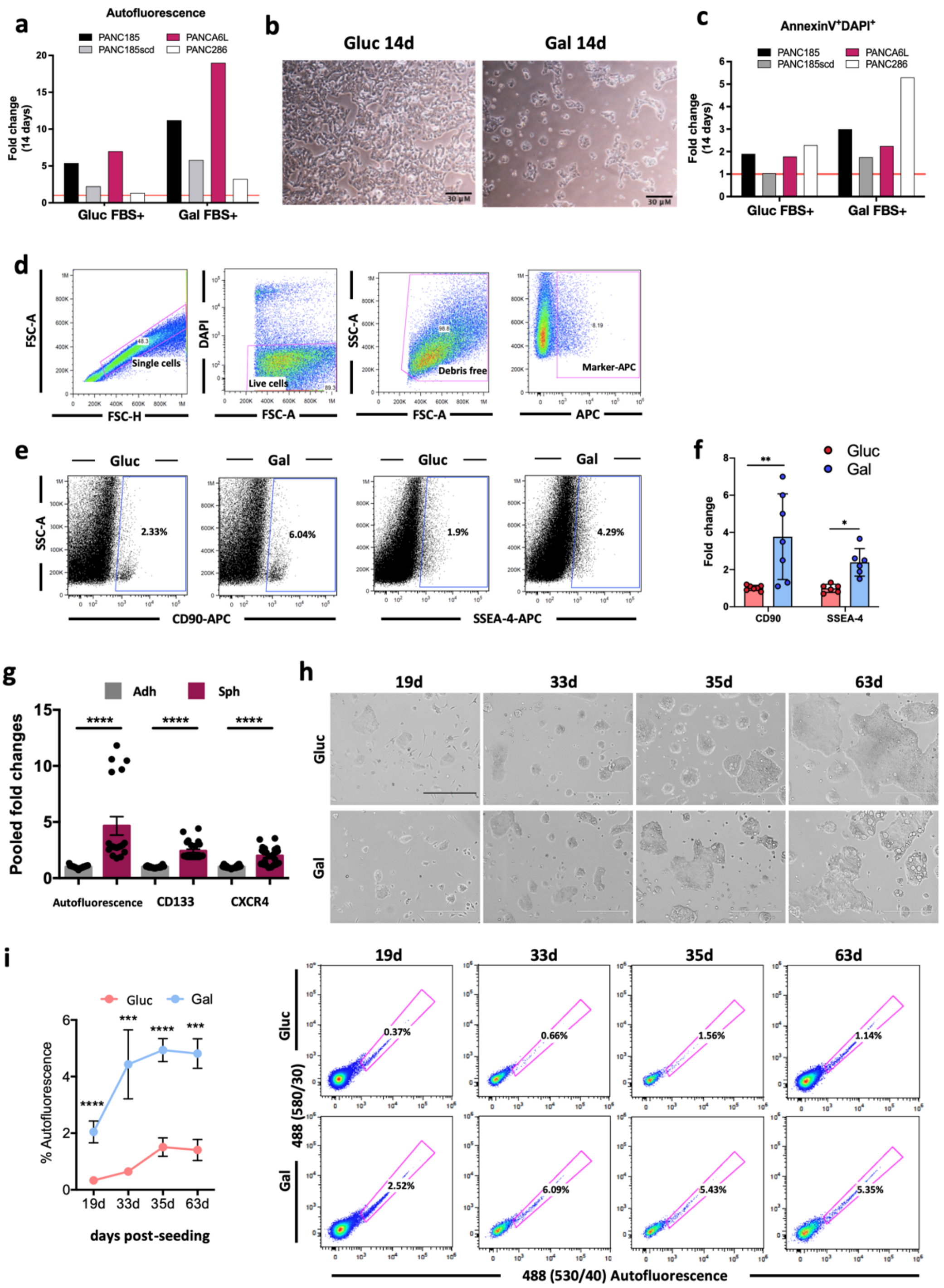

Supplementary Figure 1. Galactose-cultured cells are enriched in PaCSCs. a Analysis of the effects of fetal bovine serum (FBS)-containing media with different sources of carbon (Gluc = glucose; Gal = galactose). Fold change in the levels of the CSC autofluorescent-positive populations in PDAC primary cultures with the different indicated culture media at 14d. The red line, set at 1.0, represents basal autofluorescence in standard conditions (medium + 10% FBS and 4.5 g/L Gluc). **b** Representative images of the confluence observed in Gluc-CC and Gal-CC after 14 days. Scale bar = 30 µM. **c** Fold change in the levels of AnnexinV/$^{+}$DAPI^{+} populations in PDAC primary cultures with the different indicated culture media at 14 days. The red line, set at 1.0, represents basal AnnexinV^{+}/DAPI^{+} late apoptosis in standard conditions (medium + 10% FBS and 4.5 g/L Gluc). **d** Representative flow cytometry plots illustrating showing initial gating strategies to acquire a single cell, DAPI free (“live cell”) and debris free population, followed by an example gating

strategy used for marker detection and analysis. Additional details can be found in the Methods section. **e** Representative flow cytometric dot plots of the percentage of CD90 or SSEA-4 positive cells in PANC286 and PANC185scd, respectively, comparing Gluc-CC and Gal-CC. **f** Quantification of the levels of the different CSCs biomarkers in Gluc-CC and Gal-CC. Data are presented as fold-change ± sd. Glu was set as 1.0 (n=7 biological replicates for CD90 and n=6 biological replicates for SSEA-4; Holm-Sidak t-test). CD90 **p =0.0019 and SSEA-4 *p =0.025. **g** Quantification of the levels of the different CSCs biomarkers in adherent (Adh) versus sphere (Sph) cultures for 3 different tumors (n=4 biological replicates per tumor; Student's t-test). Data are presented pooled mean fold change ± sd. Adh was set as 1.0. **p<0.0001. **h** Light micrograph images of a PANC185 PDX tumor digested and immediately cultured in Gal- or Gluc -containing media for a total of 63 d. Scale bar = 400 µM. **i** Percent autofluorescent-positive cells ± sem detected in cultures in **h** at the indicated days post-seeding (left) and representative autofluorescent cytometry plots (right). (n=5 biological replicates; Student's t-test) ****p<0.0001; ***p=0.008; ***p=0.002.

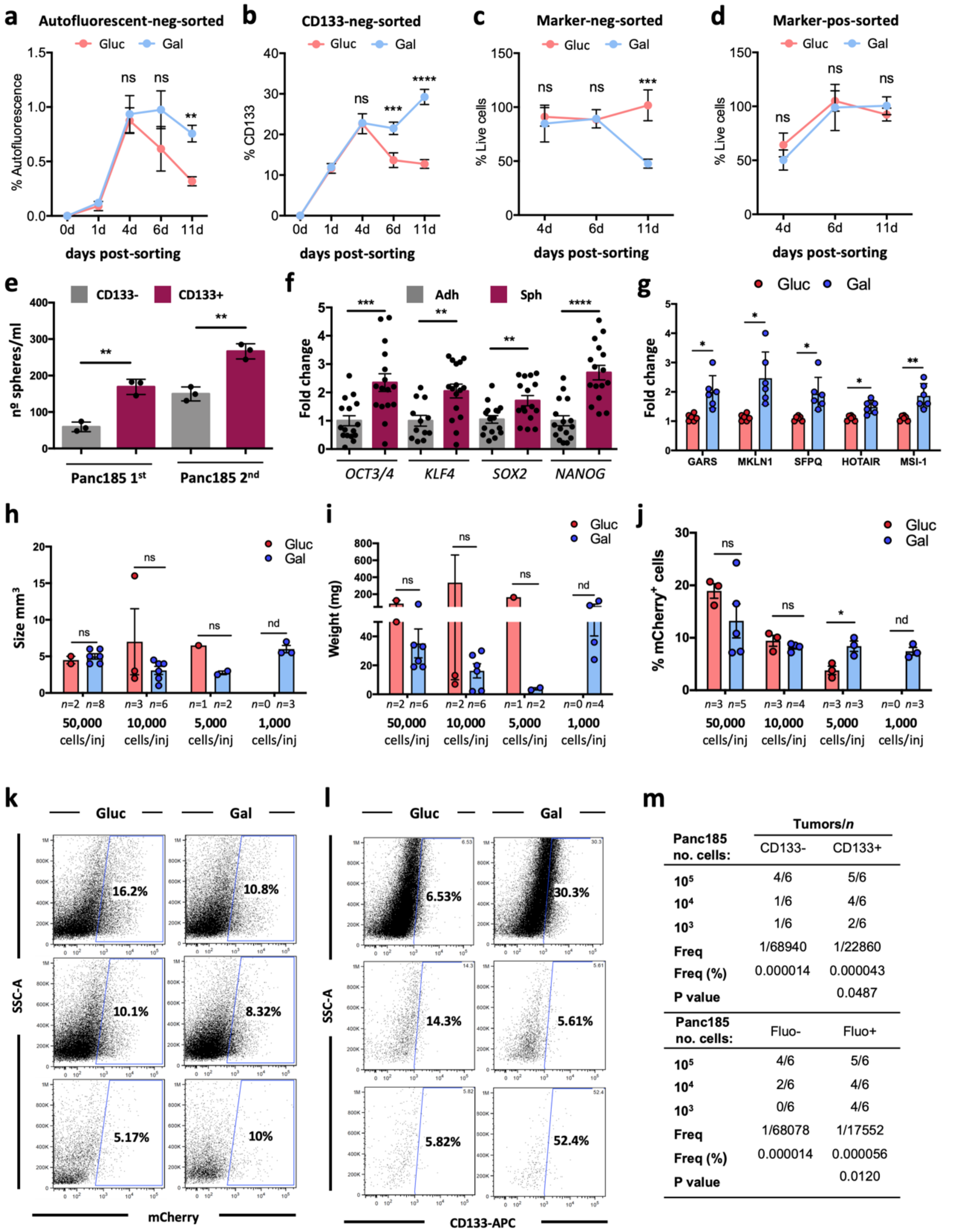

Panc185 no. cells:	Tumors/n CD133-	CD133+
10⁵	4/6	5/6
10⁴	1/6	4/6
10³	1/6	2/6
Freq	1/68940	1/22860
Freq (%)	0.000014	0.000043
P value		0.0487
**Panc185 no. cells:**	**Fluo-**	**Fluo+**
10⁵	4/6	5/6
10⁴	2/6	4/6
10³	0/6	4/6
Freq	1/68078	1/17552
Freq (%)	0.000014	0.000056
P value		0.0120

Supplementary Figure 2. Stemness validation in Gal-CC. a, b PANC185 sphere-derived cells underwent double FACS negative (neg) selection for the indicated markers. Shown are the kinetics, displayed as percent ± sd, of the reappearance of autofluorescent-positive cells **a** or CD133-positive cells **b** cells in Gal- or Gluc -containing media at the indicated times post-sorting (n=4 biological replicates from 1 experimental sorting; Student's t-test) **p=0.0024; ***p=0.0006; ****p<0.001. **c**, **d** Kinetics of the percent of live (DAPI-negative) cells ± sd for CD133-negative (neg) sorted cells **c** or CD133-positive (pos) sorted cells **d** at the indicated times post-sorting in Gal- or Gluc-containing media (n=4 biological replicates from 1 experimental sorting; Student's t-test) ***p=0.003; ns, not significant. **e** Mean number (nº) ± sd of 1st and 2nd generation (gen) spheres/mL for PANC185 CD133-

negative-sorted or Panc185 CD133-positive-sorted (n=3 biological replicates from 1 experimental sorting; Student's t-test) **p=0.0015 and 0.0020. **f** RT-qPCR analysis of different pluripotency/stemness genes in adherent (adh) versus spheres (sph) cultures for 4 different tumors. mRNA expression levels for each target gene are normalized to β-actin levels (n=4 biological replicates per tumor for all genes except for *KLF4* where n=3 biological replicates per tumor; Student's t-test). Data are presented as mean fold-change ± sd. Adh was set as 1.0. **p=0.0066; **p=0.0034; **p=0.0007; **p<0.0001. **g** RT-qPCR analysis of relative mRNA expression levels of LncRNA genes in Gluc-CC and Gal-CC. mRNA expression levels for each target gene are normalized to β-actin levels. Data are presented as pooled mean fold-change ± sd. Glu was set as 1.0 (n=3 biological replicates per 2 different tumors; Holm-Sidak t-test statistical analysis) *p=0.035; *p=0.044; *p=0.049; *p=0.03; **p=0.0013. **h** Mean tumor volume (mm^3) ± sd, **i** mean weight ± sd and **j** quantification of the mean percentage of mCherry-H2B$^+$ cells ± sd after digestion of Gluc-CC and Gal-CC tumors (n's are indicated; Holm-Sidak t-test statistical analysis). For **h**: ns=0.58; ns=0.82; ns=0.098; nd, not determined. For **i**: ns=0.067; ns=0.42; ns=0.071; nd, not determined. For **j**: ns=0.075; ns=0.46; *p=0.037; nd, not determined. **k, l** Representative flow cytometric dot plots of **k** mCherry$^+$ or **l** CD133$^+$ populations inside the mCherry$^+$ populations in Gluc-CC and Gal-CC digested tumors. **m** Number of tumors obtained/number for CSC-marker-positive versus CSC-marker-negative cells as a function of the dilutions tested. The Extreme Limiting Dilution Analysis software was used to calculate the frequency (Freq) and percent (%) of CSCs and the p value.

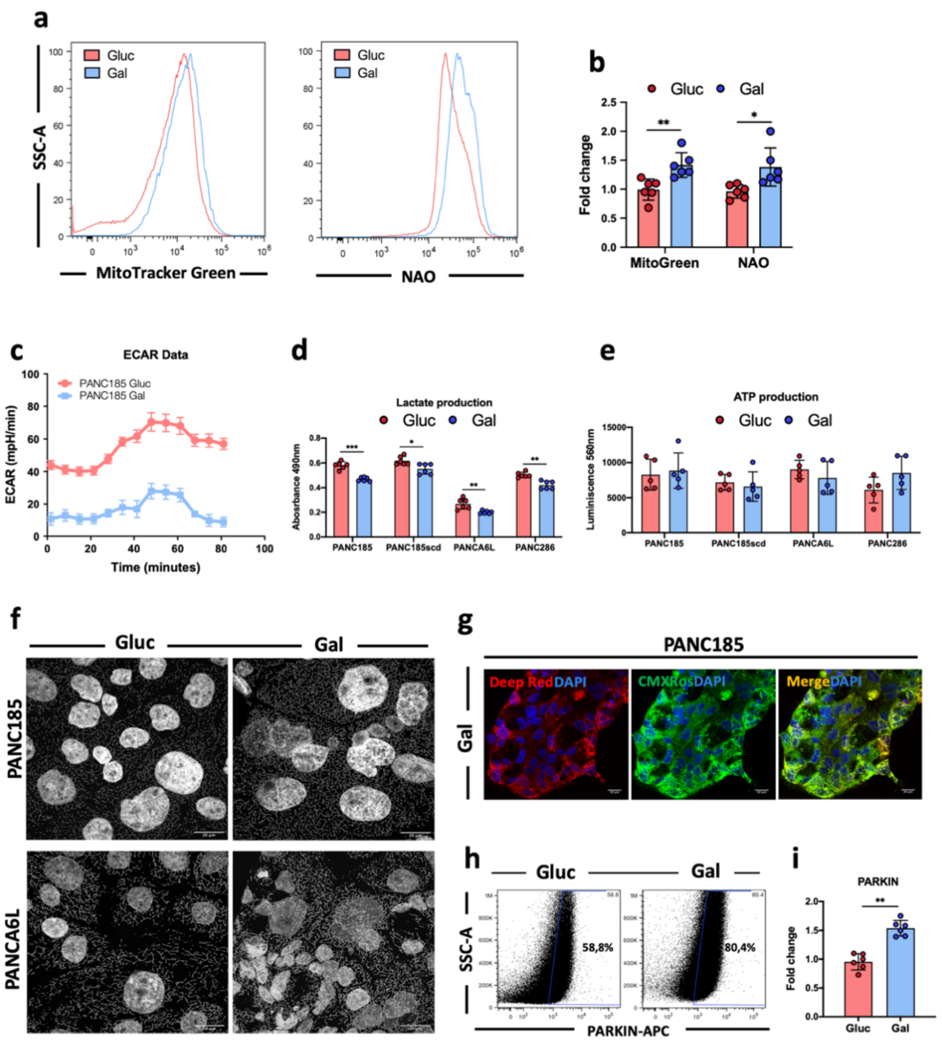

Supplementary Figure 3. Mitochondrial respiration validation. a Representative flow cytometric histograms of the mean fluorescence intensities of the mitochondrial markers MitoTracker Green FM and Nonyl Acridine Orange (NAO) in Gluc-CC and Gal-CC. **b** Quantification of mean levels ± sd of the mitochondrial markers in Gluc-CC and Gal-CC. Data are presented as fold-change. Gluc was set as 1.0. (n=6 biological replicates; Holm-Sidak t-test statistical analysis). **p=0.0036; *p=0.024 **c** Extracellular acidification rate (ECAR) measurements in Gluc-CC and Gal-CC subjected to sequential injections of glucose (Glu), Oligomycin (O) and 2-deoxyglucose (2DG). **d** Mean ± sd of lactate production levels, determined by absorbance spectrophotometry, using an ELISA as per the manufacturer's instructions, normalized to total protein content in Gluc-CC and Gal-CC (n=6 biological replicates; Holm-Sidak t-test statistical analysis). ***p=0.000014; *p=0.0139; **p=0.0039; **p=0.0049. **e** Quantification of mean levels ± sd of ATP production by luminescence spectrophotometry, normalized to total protein content in Gluc-CC and Gal-CC (n=5 biological replicates; Holm-Sidak t-test statistical analysis). No significant differences. **f** Mitochondrial network visualized using an anti-TOM20 Ab, and displayed as a skeleton network using ImageJ skeleton filter, comparing Gluc-CC with Gal-CC from PANC185 and PANCA6L. Scale bar = 20 µM. **g** Representative fluorescent images of Mitotracker DeepRedFM (red), CMXRos (green) and DAPI (blue) staining in PANC185 Gal-CC. Scale bar = 20 µM. **h** Representative flow cytometric dot plots of intracellular PARKIN staining in PANC185 Gluc-CC and Gal-CC. **i** Quantification of mean levels ± sd of PARKIN in Gluc-CC and Gal-CC. Data are presented as fold-change. Glu was set as 1.0 (n=6 biological replicates; Mann-Whitney t-test statistical analysis) **p= 0.0046.

a

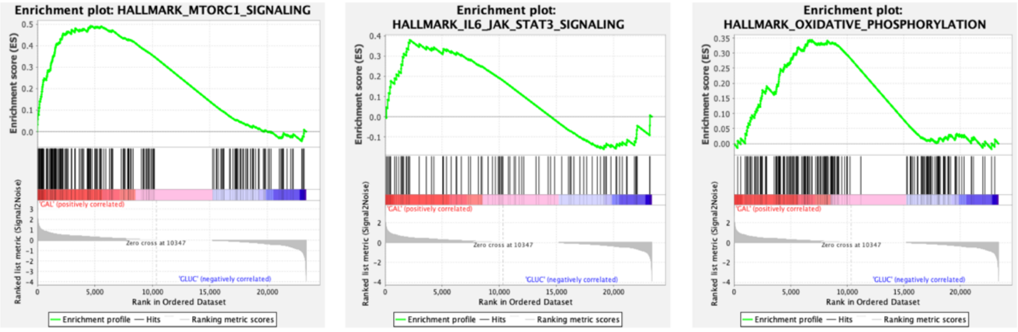

ES: 0.49170896
NES: 1.771693
Nominal p-value: 0.0
FDR q-value: 9.903652E-4
FWER p-Value: 0.002

ES: 0.38005143
NES: 1.218984
Nominal p-value: 0.09773124
FDR q-value: 0.2217476
FWER p-Value: 0.926

ES: 0.34374538
NES: 1.239324
Nominal p-value: 0.055743244
FDR q-value: 0.1975135
FWER p-Value: 0.888

b

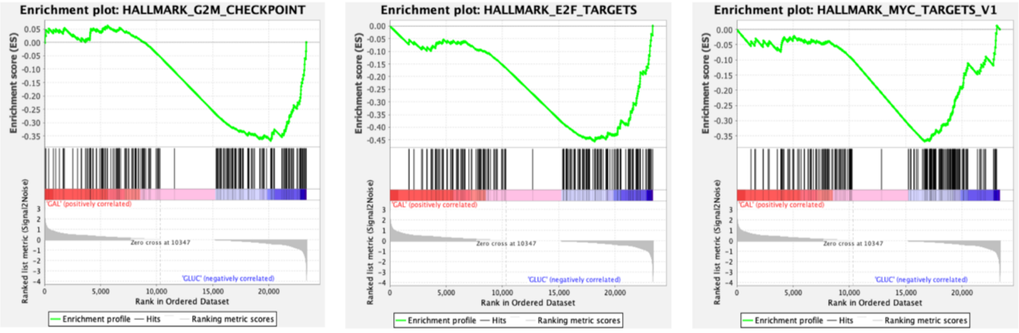

ES: -0.36779097
NES: -1.3620934
Nominal p-value: 0.011961723
FDR q-value: 0.1163448
FWER p-Value: 0.4

ES: -0.4560173
NES: -1.6867679
Nominal p-value: 0.0
FDR q-value: 0.007742744
FWER p-Value: 0.016

ES: -0.36806917
NES: -1.3545586
Nominal p-value: 0.0075376886
FDR q-value: 0.09970655
FWER p-Value: 0.421

Supplementary Figure 4. Pathway enrichment analysis. a, b Enrichment plot for pathways upregulated in PANCA6L Gal-CC: mTORC1, IL6/JAK/STAT3 and OXPHOS signaling **a,** and for pathways downregulated in PANCA6L Gal-CC: G2/M checkpoint, E2F and cMYC target genes **b**. Shown below are Enrichment scores (ES), normalized enrichment scores (NES), Nominal p-values, FDR q-values and FWER p-values.

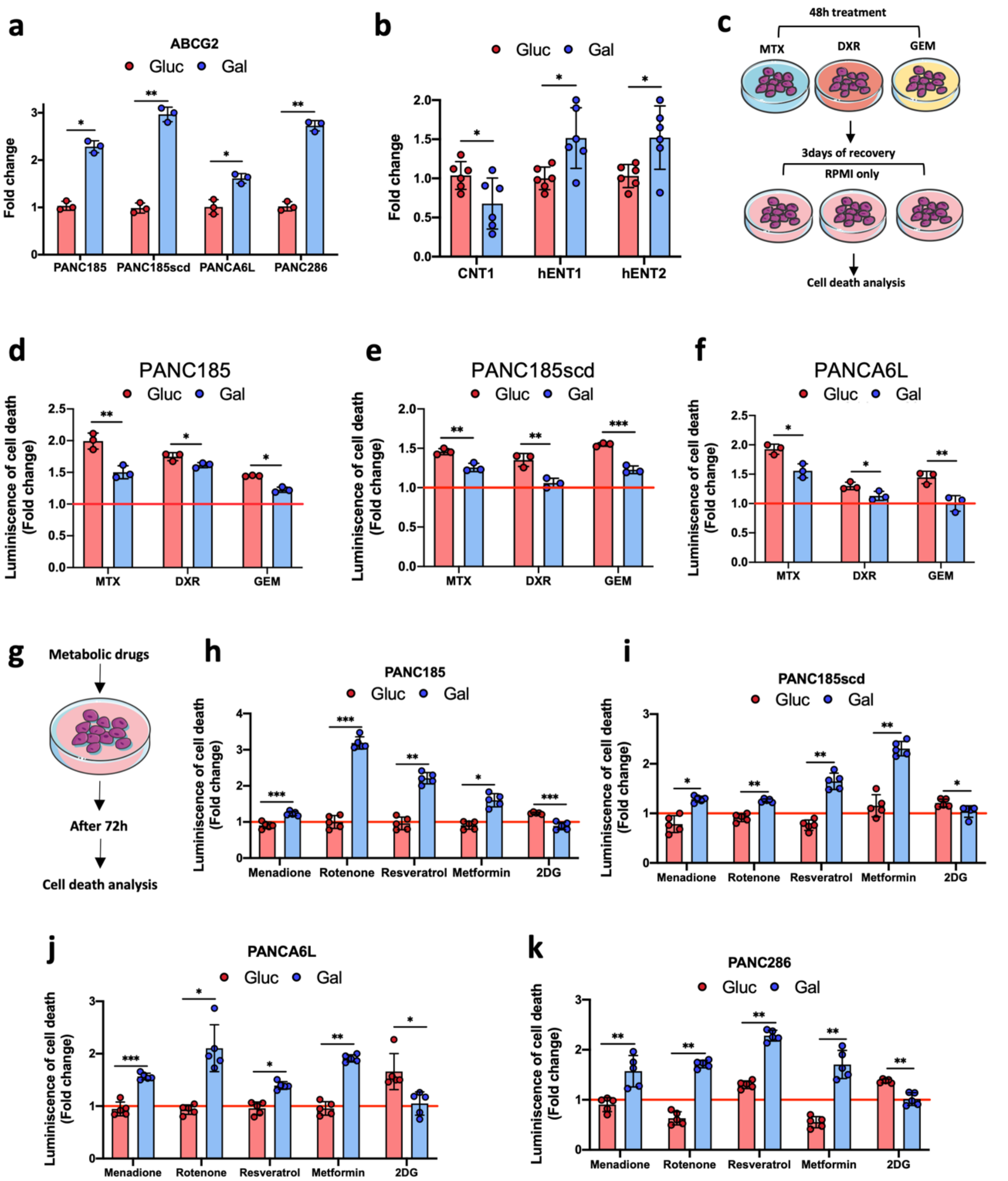

Supplementary Figure 5. Chemoresistance is enhanced in Gal-CC. a RT-qPCR analysis of mean relative mRNA expression levels ± sd of ABCG2 in Gluc-CC and Gal-CC for 4 different tumors. mRNA expression levels for each target gene are normalized to β-actin levels. Data are presented as fold-change. Gluc was set as 1.0. (n=3 biological replicates per tumor; Holm-Sidak t-test statistical analysis). *p=0.0031; **p=0.0015; *p=0.0049; **p=0.0036. **b** RT-qPCR analysis of mean relative mRNA expression levels ± sd of CNT1, hENT1 and hENT2 in Gluc-CC and Gal-CC. mRNA expression levels for each target gene are normalized to β-actin levels. Data are presented as fold-change. Gluc was set as 1.0. (n=6 biological replicates; Holm-Sidak t-test statistical analysis) *p=0.039; *p=0.012; *p=0.019. **c** Representative scheme of chemoresistance experiment with different chemotherapeutic drugs (MTX=Mitoxantrone, DXR= Doxorubicin, GEM=Gemcitabine). **d-f** Quantification of Toxilight luminescence, as a measurement of cell death, after 3 days of recovery from treatment with the indicated chemotherapeutic drugs in Gluc-CC and Gal-CC for **d** PANC185, **e** PANC185scd and **f** PANCA6L. Data are presented as mean fold-change ± sd in

luminescence, compared to luminescence measured in untreated control cultures, set as 1.0 (red line=Control) (n=3 biological replicate per tumor; Holm-Sidak t-test statistical analysis). For **d:** **p=0.0082; *p=0.0063; *p=0.037. For **e:** **p=0.0061; **p=0.0077; ***p=0.00049. For **f:** *p=0.012; *p=0.043; **p=0.010. **g** Representative scheme of chemoresistance experiment with different metabolic drugs. **h-k** Quantification of luminescence, as a measure of cell death, after 3 days of treatment with metabolic drugs comparing in Gluc-CC and Gal-CC for **h** PANC185, **i** PANC185scd, **j** PANCA6L and **k** PANC286. Data are presented as mean fold-change ± sd in luminescence, compared to luminescence measured in untreated control cultures, set as 1.0 (red line = Control) (n= 5 biological replicates per tumor; Holm-Sidak t-test statistical analysis). For **h:** ***p=0.00012; ***p=0.00002; **p=0.0021; *p=0.0087; ***p=0.000085. For **i:** *p=0.037; **p=0.0025; **p=0.0046; **p=0.008; *p=0.025. For **j:** ***p=0.00043; *p=0.0036; *p=0.041; **p=0.0052; *p=0.0013. For **k:** **p=0.0062; **p=0.0023; **p=0.0015; **p=0.0026.

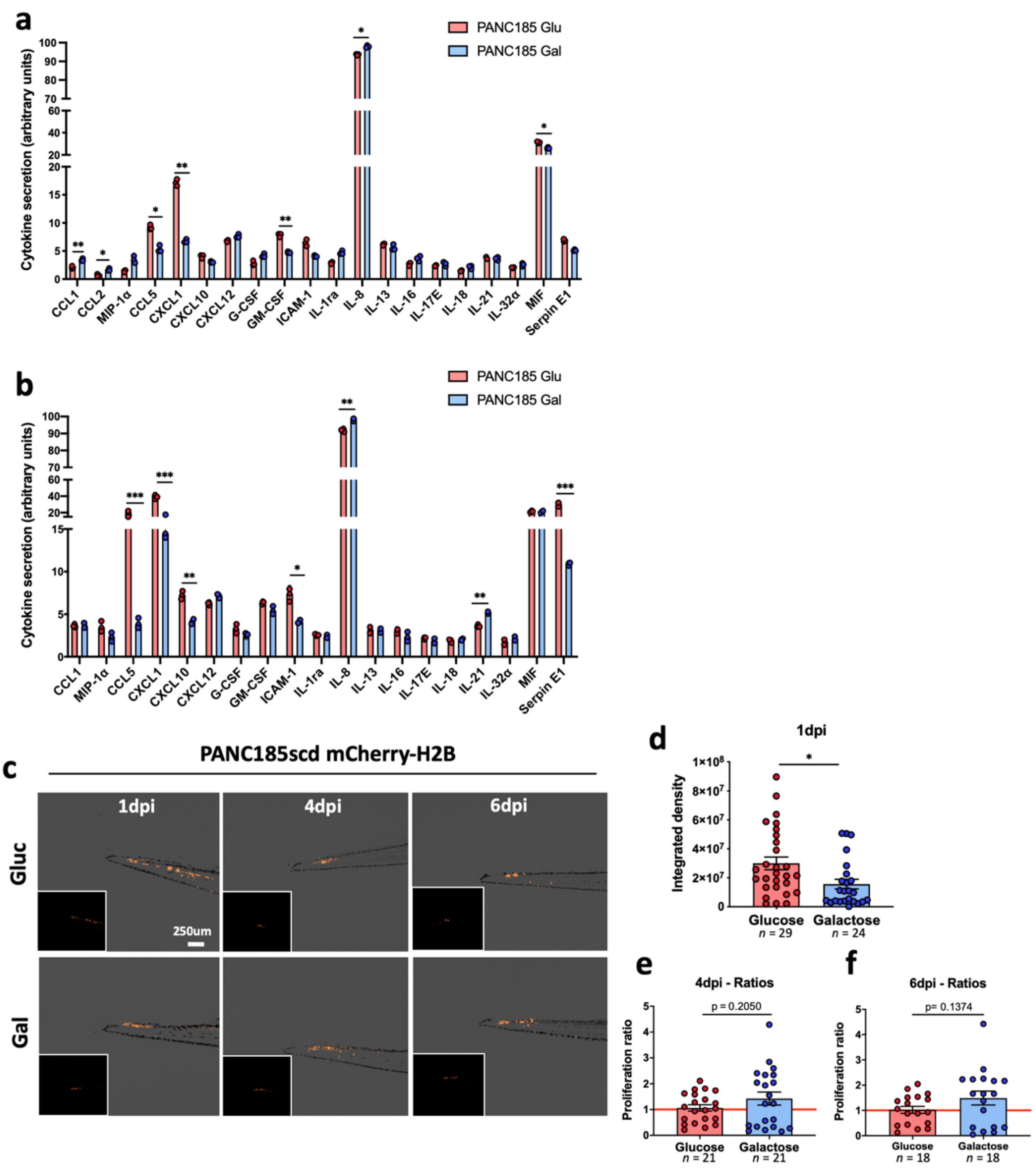

Supplementary Figure 6. Immune evasion and metastasis-invasion *in vitro* and *in vivo*. a, b Mean arbitrary units ± sd of secreted cytokines or inflammatory-related factors in Gluc-CC versus Gal-CC in **a** PANC185 and **b** PANCA6L, normalized to positive control (n=3 biological replicates per tumor; Holm-Sidak t-test statistical analysis). For **a**: **p=0.00054; *p=0.0187; *p=0.035; **p=0.0015; **p=0.0065; *p=0.023; *p=0.017. For **b**: ***p=0.00013; ***p=0.00017; **p=0.005; *p=0.028; **p=0.005; **p=0.0032; ***p=0.00047. **c** Representative images of zebrafish embryo tails taken at 1-, 4- and 6-days post-injection (dpi) with mCherry-H2B-labelled PANC185scd Gluc-CC or Gal-CC. Scale bar = 250 μM. Insets are the same image with black background. **d** Mean ± sem of the integrated density (fluorescence measured) between Gluc-CC and Gal-CC 1-day post-injection (1dpi) (n's are indicated; Student's t-test) *p=0.0149. **e, f** Mean ± sem of proliferation ratio observed between Gluc-CC and Gal-CC at **e** 4dpi and **f** 6dpi. The proliferation ratios are represented in comparison to 1dpi (red line) for both conditions (n's and p values are indicated; Student's t-test).

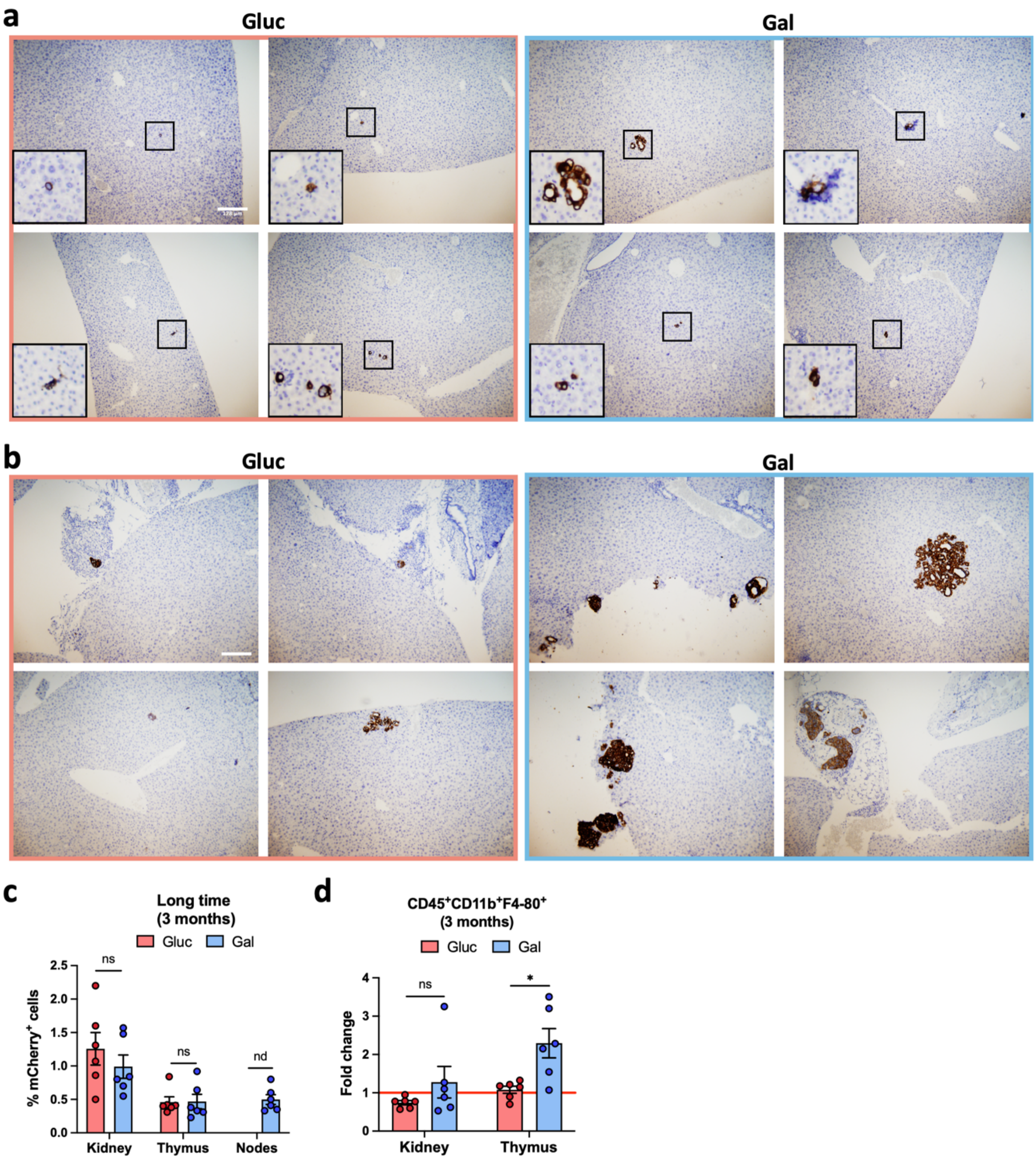

Supplementary Figure 7. Metastasis-invasion *in vivo*. a, b Representative images of human cytokeratin-19 staining in the livers of distinct animals injected with PANCA6L Gluc-CC or PANCA6L Gal-CC and extracted at 10 dpi (short time) **a** or 3 months pi (long time) **b**. Scale bar = 178 µM. For **a**, insets represent 4X zoom of positive-stained cell(s). **c** Mean fold-change ± sem of mCherry^{+} cells present in different organs analyzed (kidney, thymus and nodes) at long time (3 months post-injection of PANCA6L mCherry-H2B) comparing Gluc-CC with Gal-CC (n=1 independent experiment with a total of n=6 mice; Holm-Sidak t-test statistical analysis) ns=0.313; ns=0.672; nd, not determined. **d** Mean fold-change ± sem of the CD45^{+}CD11b^{+}F4-80^{+} population present in the different organs analyzed (kidney and thymus) at long time comparing Gluc-CC with Gal-CC. Red lines indicate baseline levels of the CD45^{+}CD11b^{+}F4-80^{+} population present in the different organs of control mice (n=6), set as 1.0 (n=1 independent experiment with a total of n=6 mice; Holm-Sidak t-test statistical analysis) ns=0.071; *p=0.022.
