# Supplementary material for: Exploiting oxidative phosphorylation to promote the stem and immunoevasive properties of pancreatic cancer stem cells
Source: Nat Commun. 2020 Oct 16;11:5265. doi: 10.1038/s41467-020-18954-z (PMC7567808; doi:10.1038/s41467-020-18954-z)
Supplement: Supplementary file 3 — Reporting Summary [file 41467_2020_18954_MOESM3_ESM.pdf]

nature research

Corresponding author(s): Bruno Sainz, Jr.

Last updated by author(s): Sep 8, 2020

Reporting Summary

Nature Research wishes to improve the reproducibility of the work that we publish. This form provides structure for consistency and transparency in reporting. For further information on Nature Research policies, see Authors & Referees and the Editorial Policy Checklist .

Statistics

For all statistical analyses, confirm that the following items are present in the figure legend, table legend, main text, or Methods section.

n/a	Confirmed	
☐	☒	The exact sample size (*n*) for each experimental group/condition, given as a discrete number and unit of measurement
☐	☒	A statement on whether measurements were taken from distinct samples or whether the same sample was measured repeatedly
☐	☒	The statistical test(s) used AND whether they are one- or two-sided<br>*Only common tests should be described solely by name; describe more complex techniques in the Methods section.*
☒	☐	A description of all covariates tested
☒	☐	A description of any assumptions or corrections, such as tests of normality and adjustment for multiple comparisons
☐	☒	A full description of the statistical parameters including central tendency (e.g. means) or other basic estimates (e.g. regression coefficient) AND variation (e.g. standard deviation) or associated estimates of uncertainty (e.g. confidence intervals)
☐	☒	For null hypothesis testing, the test statistic (e.g. *F*, *t*, *r*) with confidence intervals, effect sizes, degrees of freedom and *P* value noted<br>*Give P values as exact values whenever suitable.*
☒	☐	For Bayesian analysis, information on the choice of priors and Markov chain Monte Carlo settings
☒	☐	For hierarchical and complex designs, identification of the appropriate level for tests and full reporting of outcomes
☒	☐	Estimates of effect sizes (e.g. Cohen's *d*, Pearson's *r*), indicating how they were calculated

Our web collection on statistics for biologists contains articles on many of the points above.

Software and code

Policy information about availability of computer code

Data collection	Invitrogen™ Attune™ NxT software, version 3.1.1 was used for cytometry data collection<br>BD FACSDivaTM software v.9.0 was used for FACS data collection<br>XF96 1.4.2 Software (Agilent) for Seahorse data collection<br>The program ViewFinderTM 7.1 (Better Light, USA) was used to obtain images from the Axiovert 135 TV microscope (ZEISS, Germany)<br>The software Living image 3.2 (Perkin Elmer, Waltham, Massachusetts, USA) was used to obtain images with the IVIS-Lumina II (Caliper Life Sciences, Hopkinton, Massachusetts, USA)<br>The program Zen2009 5.5 was used to obtain images from the laser scanning confocal microscope Zeiss 710
Data analysis	For image preparation and analysis: Fiji package of ImageJ V2.0.0-rc-64/1.51s, Adobe Photoshop CS4 Extended v11.0<br>For flow cytometry image preparation and analysis: FlowJo 9.3 software (Tree Star Inc., Ashland, OR.)<br>For pathway enrichment plots and NES graphs: GSEA version 4.0.3, Broad Institute<br>For graphs preparation and analysis: Prism 8.0 for Mac, GraphPad Software, Inc<br>For OCR and ECAR analsyses: XF96 1.4.2 Software (Agilent)<br>Complete analysis of RNA-seq data was done with the pipeline: Nextpresso 1.9.2.2 (CNIO, Madrid, Spain).<br>The IncuCyte Zoom 2015A software (ESSEN BioScience, USA) was used to analyze images obtained with the IncuCyte® ZOOM System<br>The software Living image 3.2 (Perkin Elmer, Waltham, Massachusetts, USA) was used to analzye images obtained with the IVIS-Lumina II (Caliper Life Sciences, Hopkinton, Massachusetts, USA)<br>The image analysis of the injected zebrafish embryos was carried out using Quantifish software v2.1 (University College London, London, UK)

For manuscripts utilizing custom algorithms or software that are central to the research but not yet described in published literature, software must be made available to editors/reviewers. We strongly encourage code deposition in a community repository (e.g. GitHub). See the Nature Research guidelines for submitting code & software for further information.

Data

Policy information about availability of data

All manuscripts must include a data availability statement. This statement should provide the following information, where applicable:

- Accession codes, unique identifiers, or web links for publicly available datasets
- A list of figures that have associated raw data
- A description of any restrictions on data availability

RNAseq data from Gluc-CC and Gal-CC PancA6L cells, generated in this study, have been deposited in the ArrayExpress database (ref 58) at EMBL-EBI (www.ebi.ac.uk/arrayexpress) under accession number E-MTAB-9483. The source data for the main and Supplementary figures are provided as a Source Data file or can be found in the Supplementary Information file.

Field-specific reporting

Please select the one below that is the best fit for your research. If you are not sure, read the appropriate sections before making your selection.

[x] Life sciences [ ] Behavioural & social sciences [ ] Ecological, evolutionary & environmental sciences

For a reference copy of the document with all sections, see nature.com/documents/nr-reporting-summary-flat.pdf

Life sciences study design

All studies must disclose on these points even when the disclosure is negative.

Sample size	No statistical methods were used to predetermine the sample size. Sample sizes for experiments were estimated based on previous experience with a similar setup that showed significance. Published examples include (Sancho P, et al. MYC/PGC-1α Balance Determines the Metabolic Phenotype and Plasticity of Pancreatic Cancer Stem Cells. Cell Metab. 2015 Oct 6;22(4):590-605. PMID: 26365176; and Hermann PC et al. Multimodal Treatment Eliminates Cancer Stem Cells and Leads to Long-Term Survival in Primary Human Pancreatic Cancer Tissue Xenografts. PLoS One. 2013 Jun 18;8(6):e66371. PMID: 23825539, Martinez-Ordoñez A et al. Breast cancer metastasis to liver and lung is facilitated by Pit-1-CXCL12-CXCR4 axis. Oncogene. 2018 Mar;37(11):1430-1444. PMID: 29321662). Experiments involved mice were performed two to three times (as indicated) and 6-9 animals where analyzed for each experiments/ time point. Each study was designed to use the minimum number of mice required to obtain informative results (that is, quantitative data amenable to statistical analysis).<br>For experiments involving zebrafish, experiments were performed two times and a minimum of 20 embryos per experiments and per condition were injected on d0.
Data exclusions	No data were excluded
Replication	Experiments were repeated and experimental findings were reproducible. Specifically, we have included in the manuscript the following reproducibility statement "The number of biologically independent samples are indicated in the Figure legends. Repeated independent experiments per each panel with similar results are shown below. n=1 (Figs. 1a, 1b, 1c, 1d, 1e, 2b, 3d, 3f, 3h, 3i, 3k, 3l, 4a, 4d, 4g, 4h, 4i, 4j, 4k, 5c, 5e, 5f, 5h, 5i, 5k, 6e, 6g, 6h, 6k, 6l, 6m, 6n, 7g, 7i, 7k, Supplementary Figs. 1a, 1c, 1h, 1i, 2a, 2b, 2c, 2d, 2e, 2h, 2i, 2j, 2m, 4a, 4b, 3e, 5a, 5d, 5e, S5f, 6a, 6b, 6g, 6h); n=2 (Figs. 1f, 1g, 2e, 2g, 2h, 2i, 2j, 2l, 2m, 3e, 6c, 6d, 7f, 7j, Supplementary Figs. 2a, 2b, 2c, 2d, 2e, 2g, 3b, 3d, 5b, 5h, 5i, 5j, 5k); n=3 (Figs. 2f, 6b, 7b, 7c, 7d, Supplementary Figs. 1f, 3i, 6d, 6e, 6f), n=4 (Figs. 2d, 3b, Supplementary Figs. 2f)."
Randomization	Mice and zebrafish experimentations: For the in vivo experiments, mice and zebrafish were previously randomized into experimental groups. Cell line experimentation: For each experiment, the total amount of cells from one cell line required for all tested conditions were pooled and seeded randomly into different plates, pre-labeled with the treatment to be applied.
Blinding	No formal blinding was used. Tumor measurements were conducted by an independent technician who was unaware of the hypothesis or the conditions of the cells injected. Codification of the samples injected was maintained during cell injections. The analysis post mouse sacrifice was performed by an author who using the coded samples. Decoding was done post analysis. For zebra fish injections, samples were sent to 3Department of Zoology, Genetics and Physical Anthropology, Veterinary Faculty, Universidad de Santiago de Compostela, Lugo, Spain coded. Decoding was done post analysis to avoid preconceptions of the analyzing investigator. For the imaging experiments, including Confocal microscopy, where manual counting was required, samples were labeled with numbers in order to avoid preconceptions of the analyzing investigator. Automated software was used for FACS analysis, precluding bias of the investigator. If settings other than the default settings of a software was used, the parameters are specified in the respective methodology section. The remaining experiments were analyzed by western blotting or RTqPCR. In this case, codification was maintained during sample preparation. Decoding was done before loading the samples on the SDS-PAGE gel or loading the PCR plate, in order to ensure an adequate presentation of the results.

Reporting for specific materials, systems and methods

We require information from authors about some types of materials, experimental systems and methods used in many studies. Here, indicate whether each material, system or method listed is relevant to your study. If you are not sure if a list item applies to your research, read the appropriate section before selecting a response.

Materials & experimental systems

n/a	Involved in the study
☐	☒ Antibodies
☐	☒ Eukaryotic cell lines
☒	☐ Palaeontology
☐	☒ Animals and other organisms
☐	☒ Human research participants
☒	☐ Clinical data

Methods

n/a	Involved in the study
☒	☐ ChIP-seq
☐	☒ Flow cytometry
☒	☐ MRI-based neuroimaging

Antibodies

Antibodies used

WB = Western blot; FC = Flow cytometry; IF = Immunofluorescence; IHC = Immunohistochemistry
Primary Antibodies:
α-hu-CD133/1-APC, Mouse monoclonal 1:10 FC Miltenyi Biotec (Cat no. 130-090-826)
α-hu-CD24-PEVio700, Mouse monoclonal 1:10 FC Miltenyi Biotec (Cat no.130-108-381)
α-hu-CXCR4-PE, Mouse monoclonal 1:10 FC Miltenyi Biotec (Cat no.130-103-798)
α-hu-TEM8, Mouse monoclonal 1:50 FC Abcam (Cat no. ab21270)
α-hu-PD-L1-Alexa700, Mouse monoclonal 1:10 FC BioLegend (Cat no. 329952)
α-hu-CD47-APC, Mouse monoclonal 2,5:50 FC Miltenyi Biotec (Cat no 130-101-407)
α-hu-CD155-APC, Mouse monoclonal 1:10 FC Miltenyi Biotec (Cat no 130-105-906)
α-hu-CD206-FITC, Mouse monoclonal 2,5:50 FC Miltenyi Biotec (Cat no 130-095-13)
α-hu-ULBP2/5/6, Mouse monoclonal 1:50 FC R&D systems (Cat no. MAB1298)
α-hu-CD90-APC, Mouse monoclonal 2,5:50 FC Life Technologies (Cat no. A15726)
α-b-ACTIN, Mouse monoclonal 1:5000 WB ThermoFisher (Cat no.MA1-140)
α-GAPDH, Mouse monoclonal 1:5000 WB ThermoFisher (Cat no.MA5-15738)
α-hu-LC3BI/II, Rabbit monoclonal 1:500/1:100 WB/IF Sigma (Cat no. L7543)
α-hu-LAMP-1 (H43A), Mouse monoclonal 1:100 IF Santa Cruz (Cat no. sc-20011)
α-hu-PARKIN, Rabbit monoclonal 1:500/1:200 FC/IF ThermoFisher (Cat no.PA5-13398)
α-hu-SSEA4-APC, Mouse monoclonal 1:50 FC BioLegend (Cat no. 330418)
α-hu-CD44-PE, Mouse monoclonal 1:50 FC Becton Dickinson (Cat no. 550989)
TOM20, Mouse monoclonal 1:200 IF Santa Cruz (Cat no. sc-17764)
α-ms-CD45-FITC, Rat monoclonal 1:100 FC BDbioscience (Cat no. 553080)
α-ms-CD11b-PerCP Cy5.5, Rat monoclonal 1:200 FC TONBO (Cat no. 65-0112-U100)
α-ms-F4-80-PE, Recombinant human 1:100 FC Miltenyi Biotec (Cat no. 130-102-422)
α-hu- Cytokeratin 19, Mouse monoclonal 1:2000 IHC Abcam (Cat no. ab9221)
Annexin-V-FITC, 1:20, Biotium (Cat no 29001)
Secondary Antibodies
α-mouse-HRP, Sheep 1:5,000 WB Amersham (Cat no. NA9310-1ML)
α-rabbit-HRP, Donkey 1:5,000 WB Amersham (Cat no. NA9340-1ML)
α-mouse Alexa 647 Goat 1:500 IF Invitrogen (Cat. no. A32728)
α-rabbit Alexa 555 Goat 1:500 IF/FC Invitrogen (Cat no. A32732)
α-Mouse IgGs-HRP Goat 1:5000 IHC DAKO (Cat no. P0447)

Validation

All antibodies used are commercially available and validated by the manufacturers, as indicated on the respective web sites of each commercial vendor. Please refer to the commercial website of each antibody for more details. In addition, all antibodies used have been validated in published articles. Examples are given below.
- α-hu-CD133/1-APC, Cat no. 130-090-826, https://www.miltenyibiotec.com; PMIDs: 31191663, 26365176, 25841238, 22056140
- α-hu-CD24-PEVio700, Cat no.130-108-381, https://www.miltenyibiotec.com; PMIDs: 31191663
- α-hu-CXCR4-PE, Cat no.130-103-798, https://www.miltenyibiotec.com; PMIDs: 25841238, 18371365
- α-hu-TEM8, Cat no. ab21270, https://www.abcam.com/tem8atr-antibody-ab21270.html, PMIDs: 31191663
- α-hu-PD-L1-Alexa700, Cat no. 329952, https://www.biolegend.com/fr-fr/products/alexa-fluor-700-anti-human-cd279-pd-1-antibody-12365; PMIDs: 21562156
- α-hu-CD47-APC, Cat no 130-101-407, https://www.miltenyibiotec.com; PMID: 25717063
- α-hu-CD155-APC, Cat no 130-105-906, https://www.miltenyibiotec.com; PMIDs: 23980210
- α-hu-CD206-FITC, Cat no 130-095-13, https://www.miltenyibiotec.com; PMIDs: 25841238
- α-hu-ULBP2/5/6, Cat no. MAB1298, https://www.rndsystems.com/products/human-ulbp-2-5-6-antibody-165903_mab1298; PMID: 31471588
- α-hu-CD90-APC, Cat no. A15726, https://www.thermofisher.com/antibody/product/CD90-Antibody-clone-5E10-Monoclonal/A15726,; PMID: 27934856
- α-b-ACTIN, Cat no. MA1-140, https://www.thermofisher.com/antibody/product/beta-Actin-Antibody-clone-15G5A11-E2-Monoclonal/MA1-140; According to the website: This Antibody was verified by Cell treatment to ensure that the antibody binds to the antigen stated.
- α-GAPDH, Cat no. MA5-15738, https://www.thermofisher.com/antibody/product/GAPDH-Loading-Control-Antibody-clone-

GA1R-Monoclonal/MA5-15738; PMID: 27777789
- α-hu-LC3BI/II, Cat no. L7543, https://www.sigmaaldrich.com/catalog/product/sigma/l7543?lang=es&region=ES&gclid=CjwKCAjwkJj6BRA-EiwA0ZVPVm269HotFqgdCEpryG9oWDAMQLuDbJ4s5nEtaNGOPYcsDiqwbrzjmhoCbaMQAvD_BwE; This Antibody enhanced validation according to the manufacturer's webiste.
- α-hu-LAMP-1 (H43A), Cat no. sc-20011, https://www.scbt.com/p/lamp-1-antibody-h4a3; PMID: 9694904
- α-hu-PARKIN, Cat no. PA5-13398, https://www.thermofisher.com/antibody/product/Parkin-Antibody-Polyclonal/PA5-13398; PMID: 32472071
- α-hu-SSEA4-APC, Cat no. 330418, https://www.biolegend.com/en-us/products/apc-anti-human-ssea-4-antibody-13339; PMID: 29636455
- α-hu-CD44-PE, Cat no. 550989, https://www.bdbiosciences.com/eu/applications/research/t-cell-immunology/t-follicular-helper-tfh-cells/surface-markers/human/pe-mouse-anti-human-cd44-515/p/550989; PMID: 1702327
- TOM20, Cat no. sc-17764, https://www.scbt.com/p/tom20-antibody-f-10; PMID: 32472071
- α-ms-CD45-FITC, Cat no. 553080, https://www.bdbiosciences.com/eu/applications/research/stem-cell-research/cancer-research/mouse/fitc-rat-anti-mouse-cd45-30-f11/p/553080; PMID: 11062533
- α-ms-CD11b-PerCP Cy5.5, Cat no. 65-0112-U100, https://tonbobio.com/products/percp-cyanine5-5-anti-human-mouse-cd11b-m1-70; PMID: 30990169
- α-ms-F4-80-PE, Cat no 130-102-422, https://www.miltenyibiotec.com; PMIDs: 30936462
- α-hu- Cytokeratin 19, Cat no ab9221, https://www.abcam.com/cytokeratin-19-antibody-rck108-ab9221.html; PMID: 31299272
- Annexin-V-FITC, 1:20, Cat no 29001, https://biotium.com/product/annexin-v-conjugates/; PMID: 29930081
- α-mouse-HRP, Cat no. NA9310-1ML, https://www.sigmaaldrich.com/catalog/product/sigma/gena93101ml?lang=es&region=ES; PMID: 32472071
- α-rabbit-HRP, Cat no. NA9340-1ML, https://www.sigmaaldrich.com/catalog/product/sigma/gena93401ml?lang=es&region=ES; PMID: 32472071
- α-mouse Alexa 647 Cat. no. A32728, https://www.thermofisher.com/antibody/product/Goat-anti-Mouse-IgG-H-L-Highly-Cross-Adsorbed-Secondary-Antibody-Polyclonal/A32728; PMID: 31040273
- α-rabbit Alexa 555 Cat no. A32732, https://www.thermofisher.com/antibody/product/Goat-anti-Rabbit-IgG-H-L-Highly-Cross-Adsorbed-Secondary-Antibody-Polyclonal/A32732; PMID: 30842726
- α-Mouse IgGs-HRP Cat no. P0447, https://www.agilent.com/en/product/immunohistochemistry/antibodies-controls/secondary-antibodies/goat-anti-mouse-immunoglobulins-hrp-(affinity-isolated)-153239; Validated according to website

Eukaryotic cell lines

Policy information about cell lines

Cell line source(s)	PDAC patient-derived xenografts (PDAC PDX) were obtained from Dr. Manuel Hidalgo under a Material Transfer Agreement with the Spanish National Cancer Centre (CNIO), Madrid, Spain (Reference no. I409181220BSMH). PDX-derived cultures are referred to by a random number designation (e.g. Panc185, PancA6L, Panc286 or Panc185scd). 293T cells were provided by Dr. Amparo Cano (Universidad Autonoma de Madrid, Madrid, Spain).
Authentication	Cells were periodically authenticated by microsatellite analysis
Mycoplasma contamination	All cells were tested for mycoplasma every 4 weeks and were confirmed to be mycoplasma negative
Commonly misidentified lines (See ICLAC register)	No commonly misidentified cell lines were used in the study.

Animals and other organisms

Policy information about studies involving animals; ARRIVE guidelines recommended for reporting animal research

Laboratory animals	Female 8-week-old NU-Foxn1nu nude mice (Envigo, Spain) and Female 8-week-old NOD-SCID mice (in-house production - Animal Facility, Instituto de Investigaciones Biomédicas "Alberto Sols" CSIC-UAM, Madrid, Spain). Mice were housed according to the following guidelines: a 12 h light/12 h dark cycle, with no access during the dark cycle; temperatures of 65-75°F (~18-23°C) with 40-60% humidity; a standard diet with fat content ranging from 4% to 11; sterilized water was accessible at all times; for handling, mice were manipulated gently and as little as possible; noises, vibrations and odors were minimized to prevent stress and decreased breeding performance; and enrichment was always used per the facility's guidelines to help alleviate stress and improve breeding. These details are also included in the article.<br>Zebrafish embryos (0 hours post-fertilization) were used in this study, obtained by mating adult zebrafish (species: Danio rerio, wild-type). Embryos were maintained in 30L tanks with a ratio of 1 fish per liter of water, with 14h/10h light/dark cycle and a temperature of 28.5°C according to Cabezas-Sainz, P. et al. Improving zebrafish embryo xenotransplantation conditions by increasing incubation temperature and establishing a proliferation index with ZFtool. BMC Cancer 18, 3 (2018).
Wild animals	The study did not involve wild animals.
Field-collected samples	The study did not involve samples collected from field.
Ethics oversight	Mice were housed according to institutional guidelines and all experimental procedures were performed in compliance with the institutional guidelines for the welfare of experimental animals approved by the Universidad Autónoma de Madrid Ethics Committee (CEI 60-1057-A068 and CEI 103-1958-A337) and La Comunidad de Madrid (PROEX 335/14 and PROEX 294/19) and in accordance with the guidelines for Ethical Conduct in the Care and Use of Animals as stated in The International Guiding

	Principles for Biomedical Research involving Animals, developed by the Council for International Organizations of Medical Sciences (CIOMS).<br>All the procedures used in the zebrafish in vivo experiments as well as fish care were performed in agreement with the Animal Care and Use Committee of the University of Santiago de Compostela and the standard protocols of Spain (Directive 2012-63-UE).

Note that full information on the approval of the study protocol must also be provided in the manuscript.

Human research participants

Policy information about studies involving human research participants

Population characteristics	For the human healthy serum samples used in this study, no identifying information was obtained or used.
Recruitment	Patients were not recruited for this specific study, but rather samples were retrospectively obtained from collections already established by the the BioBank Hospital Ramón y Cajal-Instituto Ramón y Cajal de Investigaciones Sanitarias (IRYCIS) (PT13/0010/0002, Instituto de Sanidad Carlos III, ISCIII Biobank Register No. B.0000678). Samples used are non-identifiable, as detailed above.
Ethics oversight	Serum samples were provided by the BioBank Hospital Ramón y Cajal-Instituto Ramón y Cajal de Investigaciones Sanitarias (IRYCIS) (PT13/0010/0002), integrated in the Spanish National Biobanks Network (Instituto de Sanidad Carlos III, ISCIII, Biobank Register No. B.0000678). Samples were processed following standard operating procedures with the appropriate approval of the IRYCIS Ethical and Scientific Committees (Control no. No. Control: DE-BIOB-73 AC65, RG.BIOB-57, and RG.BIOB-54), with informed consent and according to Declaration of Helsinki principles.

Note that full information on the approval of the study protocol must also be provided in the manuscript.

Flow Cytometry

Plots

Confirm that:

- [x] The axis labels state the marker and fluorochrome used (e.g. CD4-FITC).
- [x] The axis scales are clearly visible. Include numbers along axes only for bottom left plot of group (a 'group' is an analysis of identical markers).
- [x] All plots are contour plots with outliers or pseudocolor plots.
- [x] A numerical value for number of cells or percentage (with statistics) is provided.

Methodology

Sample preparation	Cells were trypsinzed, blocked with Flebogamma and resuspended in Flow buffer [1X PBS; 3% FBS (v/v); 3mM EDTA (v/v)] with or without the appropriate diluted fluorescently-tagged antibody against the antigen of choice or with an IgG control.
Instrument	Invitrogen™ Attune™ NxT (cytometry), FACS Vantage SE Flow Cytometer (sorting)
Software	Invitrogen™ Attune™ NxT software, version 3.1.1 was used for cytometry data collection, BD FACSDiVa software was used for sorting data collection, and FlowJo 9.3 software (Tree Star Inc., Ashland, OR) was used for flow cytometry image preparation and analysis.
Cell population abundance	Cancer stem cells represent a small percentage of the total cell population (<10%) and often times purity is not confirmed due to low cell numbers recovered post sorting. When confirmed using the Invitrogen™ Attune™ NxT, purity is typically between 80-90%.
Gating strategy	Generally, cells was first gated on FSC-Area/FSC-Height to remove aggregates. Dead cells were removed by gating in DAPI-negative cells versus FSC-Area. Debris free, live, single cells were gated using FSC-Area and SSC-Area. Surface antigen gating was performed on the live, single, debris free cell population. Gates were determined based on negative controls, unstained controls or IgG controls. A figure exemplifying all of the gating strategies used and controls is provided in Supplementary Figure 1d.

- [x] Tick this box to confirm that a figure exemplifying the gating strategy is provided in the Supplementary Information.
